# Ectopic Bone Formation by Mesenchymal Stem Cells Derived from Human Term Placenta and the Decidua

**DOI:** 10.1371/journal.pone.0141246

**Published:** 2015-10-20

**Authors:** Gina D. Kusuma, Danijela Menicanin, Stan Gronthos, Ursula Manuelpillai, Mohamed H. Abumaree, Mark D. Pertile, Shaun P. Brennecke, Bill Kalionis

**Affiliations:** 1 Department of Obstetrics and Gynaecology, Royal Women’s Hospital, The University of Melbourne, Parkville, Victoria, Australia; 2 Pregnancy Research Centre, Department of Perinatal Medicine, Royal Women’s Hospital, Parkville, Victoria, Australia; 3 Mesenchymal Stem Cell Laboratory, Faculty of Health Sciences, School of Medical Sciences, University of Adelaide, Adelaide, Australia; 4 Colgate Australian Clinical Dental Research Centre, School of Dentistry, University of Adelaide, Adelaide, Australia; 5 Centre for Genetic Diseases, Monash Institute of Medical Research-Prince Henry’s Institute of Medical Research and Monash University, Clayton, Victoria, Australia; 6 King Abdullah International Medical Research Center/ King Saud Bin Abdulaziz University for Health Sciences, College of Science and Health Professions, King Abdulaziz Medical City-National Guard Health Affairs, Riyadh, Kingdom of Saudi Arabia; 7 Victorian Clinical Genetics Services (VCGS), Murdoch Children’s Research Institute, Royal Children's Hospital, Parkville, Victoria, Australia; 8 Department of Paediatrics, Royal Children’s Hospital, The University of Melbourne, Parkville, Victoria, Australia; Osaka University, JAPAN

## Abstract

Mesenchymal stem cells (MSCs) are one of the most attractive cell types for cell-based bone tissue repair applications. Fetal-derived MSCs and maternal-derived MSCs have been isolated from chorionic villi of human term placenta and the *decidua basalis* attached to the placenta following delivery, respectively. Chorionic-derived MSCs (CMSCs) and decidua-derived MSCs (DMSCs) generated in this study met the MSCs criteria set by International Society of Cellular Therapy. These criteria include: (i) adherence to plastic; (ii) >90% expression of CD73, CD105, CD90, CD146, CD44 and CD166 combined with <5% expression of CD45, CD19 and HLA-DR; and (iii) ability to differentiate into osteogenic, adipogenic, and chondrogenic lineages. *In vivo* subcutaneous implantation into SCID mice showed that both bromo-deoxyuridine (BrdU)-labelled CMSCs and DMSCs when implanted together with hydroxyapatite/tricalcium phosphate particles were capable of forming ectopic bone at 8-weeks post-transplantation. Histological assessment showed expression of bone markers, osteopontin (OPN), osteocalcin (OCN), biglycan (BGN), bone sialoprotein (BSP), and also a marker of vasculature, alpha-smooth muscle actin (α-SMA). This study provides evidence to support CMSCs and DMSCs as cellular candidates with potent bone forming capacity.

## Introduction

Mesenchymal stem cells (MSCs), which are also referred to as multipotent stromal cells, are found in many tissues. MSCs are capable of multipotent differentiation, allowing them to contribute to bone regeneration and repair since MSCs can readily differentiate into osteocytic lineages [1]. Moreover, MSCs are readily isolated, their numbers can be greatly expanded in culture, cryopreserved for later use, and importantly, they display low immunogenicity, are immunomodulatory and have a good safety profile. According to the International Society for Cellular Therapy (ISCT), MSCs must; (i) adhere to untreated plastic surfaces; (ii) express CD105, CD73, and CD90 but not CD34, CD14, CD19, CD11b, CD79α or HLA-DR, and (iii) differentiate into osteogenic, adipogenic and chondrogenic lineages *in vitro* [2].

The human term placenta is an abundant, readily accessible and non-controversial source of MSCs. MSCs have been isolated from fetal derived placental tissues including the amnion, chorion and chorionic villi, and from maternal derived tissues that are attached to placental tissue following delivery i.e. the *decidua parietalis* and *decidua basalis* [3–7]. The peripheral region of the placenta on the maternal side that is in contact with the uterine wall (called the basal plate) comprises the chorionic villi on one side, and maternal *decidua basalis* on the other. Following delivery of the placenta, the *decidua basalis* remains attached to the maternal side of the placenta. Thus, careful preparation and characterization needs to be carried out to confirm the maternal origins of *decidua basalis* MSCs (DMSCs) and the fetal origins of the chorionic villous MSCs (CMSCs). As such, in addition to the criteria stipulated by the ISCT, Parolini et al. proposed that CMSCs should have <1% maternal cells in the population [8, 9]. The significant issue of the presence of maternal cells in human placental MSCs cultures was reviewed recently [10]. Therefore, the first aim of this study was to isolate and characterize CMSCs and DMSCs according to the criteria described above and to confirm the respective fetal and maternal origins of these cells. This characterization was an essential prerequisite to the use of CMSCs and DMSCs for *in vivo* assays.

While studies have reported osteogenesis by CMSCs and DMSCs *in vitro*, bone formation *in vivo* has not been investigated. Such studies are essential for evaluating the functional capacity of CMSCs and DMSCs and their potential for clinical applications. Therefore, we initiated the study using a mouse model of ectopic bone formation to explore the possibility that isolated human CMSCs and DMSCs were capable of regenerating ectopic bone-like structure *in vivo*.

The orthotopic bone formation assay is commonly used to study osteogenesis *in vivo*. Compared to the orthotopic assay, the ectopic bone forming assay has unique advantages since there is no requirement for bone cytokine stimulation and cell-to-cell interaction with endogenous bone-forming cells [11]. In addition, a variety of ectopic locations can be used for cell implantation, including subcutaneous and intramuscular sites and the kidney capsule [11]. Subcutaneous implantation is the simplest experimental model of ectopic bone formation. Mouse models are preferable and most widely used due to their low cost, loose skin folds that can accommodate large implants, and the availability of immunodeficient mice that will accept implanted human cells. Another important consideration is the lack of naturally occurring bone-forming stem cells within the intradermal compartment and therefore newly-formed bone can be confidently attributed to the exogenous stem cells. The most pertinent concern regarding subcutaneous implantation is the lack of robust bone growth which may be due to poor blood flow. However, subcutaneous bone formation can be stimulated by incorporating hydroxyapatite and tricalcium phosphate (HA/TCP) together with stem cells. HA/TCP are currently used as bone graft substitutes, are biocompatible and form bonds between bone and ceramic implants [12]. The second and principal aim of this study was to evaluate *in vivo* bone formation capacity of CMSCs and DMSCs following subcutaneous implantation together with HA/TCP.

## Materials and Methods

### Tissue collection

Placental samples were collected from healthy women with normal pregnancies following elective Caesarean section or vaginal delivery at term (n = 6). The placental tissue had no obvious signs of calcification, infarcts or meconium staining. Exclusion criteria were women who smoked or had a twin or triplet pregnancy, drug dependency, intrauterine infection, prolonged rupture of the fetal membranes or placental abruption. Informed written consent was obtained from all participants before delivery. The study was approved by the Royal Women’s Hospital Human Research Ethics Committee.

### Isolation of CMSCs

CMSCs were isolated using the explant method as described previously [7] with the following modifications. Briefly, an incision was made through the fetal membranes near the umbilical cord insertion site and 1 g of chorionic villous tissue was obtained from approximately 1–2 cm below the chorionic plate. Pieces of chorionic tissue with typical villous morphology were cleaned with a 21 gauge needle under a dissecting microscope to remove non-villous tissue. Cleaned villi were finely diced and digested in 0.25% trypsin for 40 min at 37°C. The trypsin was inactivated with FBS and tissues were washed in PBS. The digested villi were cultured in Amniomax C100 complete medium (Life Technologies) in 25 cm^2^ tissue culture flasks maintained at 37°C in a humidified 5% CO_2_ incubator. After 7 days, villous tissues were removed from the flask and the adherent cells arising from the explants (P0 cells) were grown until at least 80% confluent before expanding to reach P5.

### Isolation of DMSCs

We have previously reported the isolation of DMSCs from the *decidua basalis* adhering onto the maternal side of the placenta [13]. About eight grams of placental tissue was dissected from the basal plate, washed four times in PBS, finely minced and digested in trypsin (0.25%; Life Technologies, CA, USA) and DNAse 1 (50 μg/mL; Worthington, NJ, USA) at 4°C overnight. Fetal bovine serum (FBS; Thermo Scientific, MA, USA) was added to inactivate the trypsin and the digest was centrifuged at 200*g* for 5 min. The pelleted tissue was digested in type 1 collagenase (10 mg/mL; Worthington) and DNAse 1 (50 μg/mL, Worthington) for 30 min at 37°C and strained through a 100 μm stainless steel sieve. The filtrate was layered over Histopaque (Sigma-Aldrich, MO, USA) and separated by density gradient centrifugation at 400*g* for 30 min. Mononuclear cell layers containing the DMSCs were aspirated and centrifuged at 200*g* for 5 min. DMSCs were maintained in α-MEM medium (Sigma-Aldrich) with 10% FBS, penicillin/streptomycin (100 U/mL and 100 mg/mL, respectively; Life Technologies) and 2 mM L-glutamine (Sigma-Aldrich). P0 DMSCs were passaged after reaching 80% confluence and cells were expanded up to P5.

### Fluorescence in situ hybridisation (FISH)

FISH was used to determine whether the DMSCs were maternal and the CMSCs fetal in origin as described elsewhere [6, 13]. Briefly, term placentae delivered from pregnancies carrying male babies (n = 3) were used to prepare DMSCs and CMSCs. Cells were lifted with TrypLE Express, washed in Hank’s buffered saline solution (HBSS; Life Technologies) and placed on poly-L-lysine coated glass slides (Thermo Scientific). After fixing in 3:1 methanol to acetic acid solution at room temperature (RT), cells were hybridized with labelled chromosome X (Spectrum Green) and chromosome Y (Spectrum Orange) probes (Abbott Molecular, MO, USA). Approximately 200 cells per slide were examined.

### Flow cytometry

To determine whether expanded cells expressed positive and negative markers characteristic of MSCs, cells were analyzed by flow cytometry for CD73, CD105, CD90, CD146, CD44 and CD166 and the absence of CD45, CD19 and HLA-DR. Cells were incubated with each of the primary antibodies or equivalent concentrations of matched isotype controls (Table 1) as described previously [13]. Cells were then washed in HBSS containing 2% FBS and centrifuged at 1000 rpm for 5 min. Cell pellets were resuspended in 500 μL of HBSS with 2% FBS and 1 μg/mL DAPI (Sigma-Aldrich). The cells were analyzed on a LSRII flow cytometer with FACS Diva software (BD Biosciences, CA, USA).

**Table 1:** Antibodies used for characterizing CMSCs and DMSCs by flow cytometry.

Antibody^a^	Conjugate	Clone	Volume / 100μl	Manufacturer
CD45	APC-Cy7	2D1	1μl	BD Biosciences
CD73	PE	AD2	1μl	BD Biosciences
CD105	APC	SN6	0.5μl	eBioscience
CD90	PE	5E10	0.25μl	BD Biosciences
CD146	PE	P1H12	1μl	BD Biosciences
CD44	PE	G44-26	1μl	BD Biosciences
CD166	PE	3A6	1μl	BD Biosciences
HLA-DR	APC	G46-6	1μl	BD Biosciences
CD19	APC-Cy7	SJ25C1	0.25μl	BD Biosciences
IgG1 isotype control	PE	MOPC-21	1μl	BD Biosciences
IgG2a isotype control	APC	G155-178	1μl	BD Biosciences
IgG1 isotype control	APC-Cy7	MOPC-21	0.25μl	BD Biosciences

^a^ anti-human antibodies raised in mice.

### *In vitro* differentiation into mesenchymal lineages

Differentiation of DMSCs and CMSCs into adipogenic, osteogenic, and chondrogenic lineages was assessed *in vitro*. *In vitro* differentiation was carried out with bullet kits as described [5, 6, 13]. Adipogenic and osteogenic differentiation was carried out in Mesencult basal medium together with the respective differentiation supplements according to manufacturer’s instructions (Stem Cell Technologies, BC, Canada). Chondrogenic differentiation was carried in DMEM/F12 medium (Life Technologies) with 1% ITS and chondrogenic supplements (both from R&D Systems, MN, USA) according to the manufacturer’s instructions. Cells were stained with Oil Red O solution, Alizarin Red solution and Safranin O (Sigma-Aldrich) to visualize adipogenesis, osteogenesis and chondrogenesis respectively.

### *In vivo* ectopic bone formation assay

CMSCs and DMSCs (n = 3 each) were expanded to reach approximately 1x10^7^ cells per sample (P2-P3). Approximately 2x10^6^ MSCs from each donor were mixed with 40 mg hydroxyapatite/tricalcium phosphate (HA/TCP) ceramic particles (Zimmer Inc., IN, USA) and then subcutaneously transplanted into the dorsal surface of eight-week-old SCID mice (National Institutes of Health-bg-nu-xid; Harlan Sprague-Dawley, IN, USA) as described previously [14, 15]. Each mouse received two implants. These procedures were performed in accordance with guidelines of an approved small-animal protocol (South Australia Pathology Animal Ethics Committee #139/09). After 8 weeks, the implants were removed, fixed in 10% formalin overnight at 4°C, and then decalcified for 2 weeks in 0.5 M EDTA, prior to paraffin embedding. For histological analysis, 5 μm sections of the implants were prepared and stained with haematoxylin and eosin (H&E). Expression of the specific osteogenesis markers osteocalcin (OCN), osteopontin (OPN), biglycan (BGN), and bone sialoprotein (BSP), were analyzed by immunohistochemistry using previously published methods [16]. The implanted cells were labelled with BrdU at 24 and 48 hrs prior to implantation to evaluate the localization of the transplanted cells. Immunohistochemical staining using an anti-BrdU antibody was carried out as previously described [17].

## Results

### Isolation and expansion of CMSCs and DMSCs

**Fig 1:**
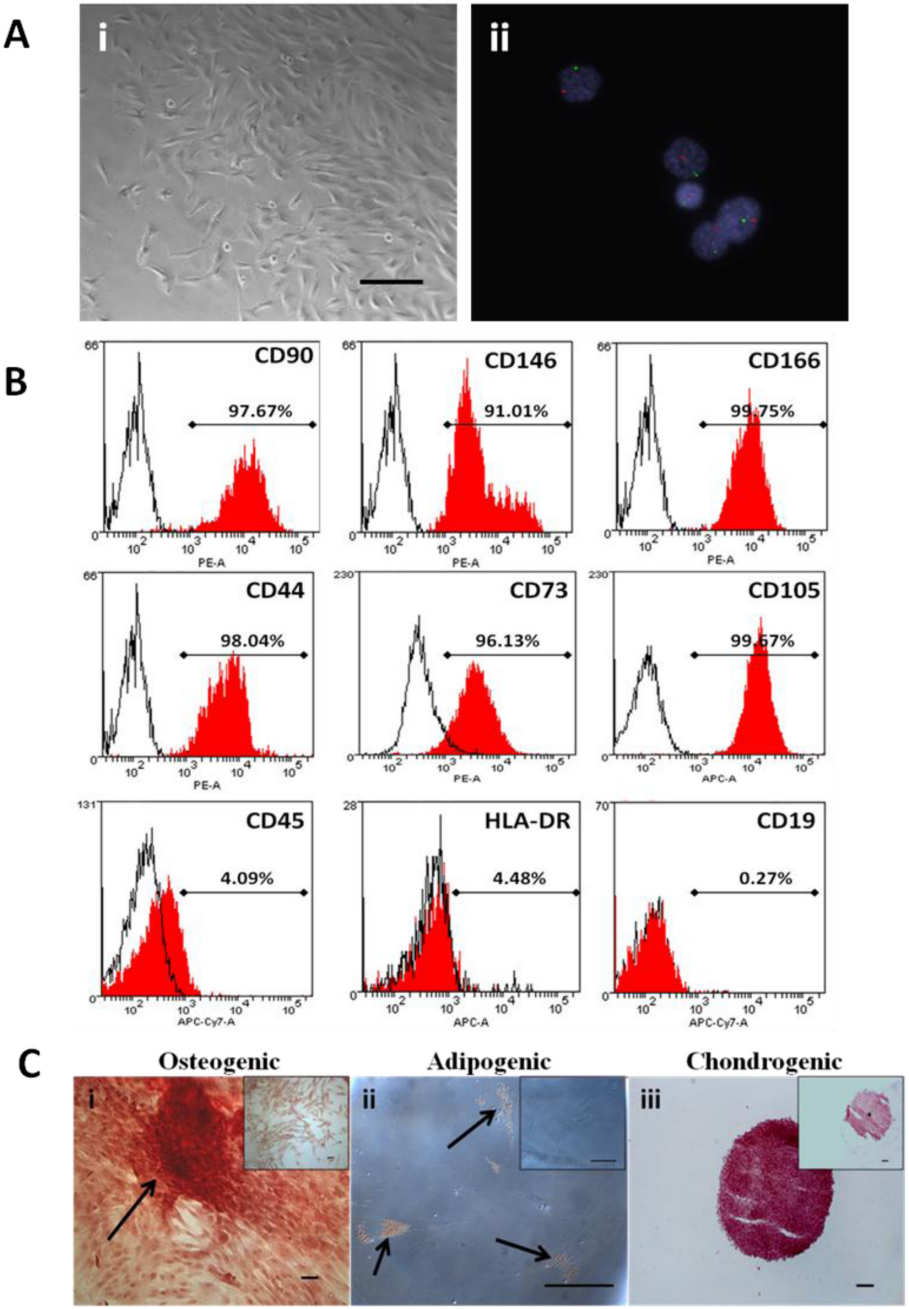
CMSC phenotypic characterization. A. (i) Bright field microscopy image of CMSCs at P0. Magnification is 100X and scalebar is 100 μm. (ii) CMSCs from placentae of male newborns were analyzed using interphase FISH on MSC nuclei. CMSCs showed one chromosome X (Spectrum Green) and one chromosome Y (Spectrum Orange) signals. Cell nuclei were stained blue with DAPI. Magnification is 630X. B. Primary CMSCs cell surface markers expression. Histograms of representative primary CMSC at P3 depicting the expression of CD90, CD146, CD166, CD44, CD73, CD105, CD45, HLA-DR, and CD19. The red histogram shows the MSC marker antibody staining while the white histogram shows the corresponding isotype control antibody staining. PE: phycoerythrin dye, APC: allophycocyanin dye, APC-Cy7: allophycocyanin-Cy7 dye. C. Representative photomicrographs showing CMSCs differentiation into mesenchymal lineages. (i) Osteogenic differentiation, Alizarin Red staining in cells after 5weeks growth in osteogenic induction medium. Arrows show calcium depositions. (ii) Adipogenic differentiation, Oil Red O staining in cells after 14 days growth in adipogenic induction medium. Arrows show fat droplets. (iii) Chondrogenic differentiation, Safranin O staining for proteoglycans depositions in cells after 21 days growth in chondrogenic induction medium. Inset shows control uninduced CMSCs. Scalebar is 100 μm.

**Fig 2:**
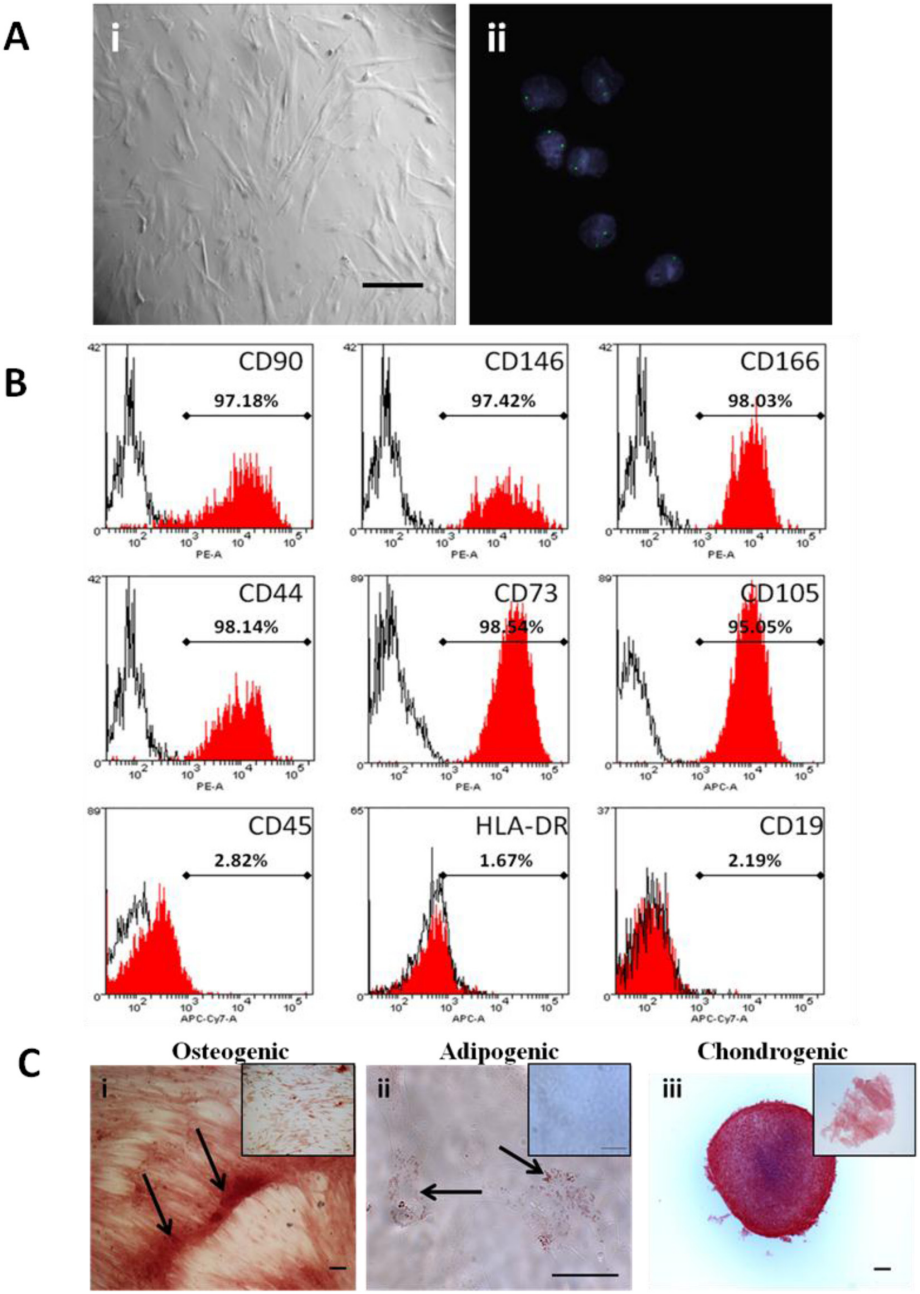
DMSC phenotypic characterization. A. (i) Bright field microscopy image of DMSCs at P0. Magnification is 100X and scalebar is 100 μm. (ii) DMSCs from placentae of male newborns were analyzed using interphase FISH on MSC nuclei. DMSCs showed two X chromosomes (Spectrum Green) signals. Cell nuclei were stained blue with DAPI. Magnification is 630X. B. Primary DMSCs cell surface markers expression. Histograms of representative primary DMSC at P3 depicting the expression of CD90, CD146, CD166, CD44, CD73, CD105, CD45, HLA-DR, and CD19. The red histogram shows the MSC marker antibody staining while the white histogram shows the corresponding isotype control antibody staining. PE: phycoerythrin dye, APC: allophycocyanin dye, APC-Cy7: allophycocyanin-Cy7 dye. C. Representative photomicrographs showing DMSCs differentiation into mesenchymal lineages. (i) Osteogenic differentiation, Alizarin Red staining in cells after 5weeks growth in osteogenic induction medium. Arrows show calcium depositions. (ii) Adipogenic differentiation, Oil Red O staining in cells after 14 days growth in adipogenic induction medium. Arrows show fat droplets. (iii) Chondrogenic differentiation, Safranin O staining for proteoglycans depositions in cells after 21 days growth in chondrogenic induction medium. Inset shows control uninduced DMSCs. Scalebar is 100 μm.

CMSCs were isolated using the explant method [5, 7]. CMSCs migrated from the explants approximately 7 days after plating. P0 and expanded CMSCs exhibited the characteristic the fibroblast-like morphology of MSCs (Fig 1Ai). DMSCs isolated from the *decidua basalis* attached to chorionic villi of term placentae adhered onto tissue culture flasks within 24 h of plating. Consistent with previous findings [18–20], P0 DMSCs were initially heterogeneous and became more homogeneous following expansion and had the fibroblastic morphology characteristic of MSCs (Fig 2Ai). Given that CMSCs and DMSCs were morphologically indistinguishable after passaging, it was crucial that CMSCs and DMSCs used in these experiments were well-characterized with respect to their surface markers expression, origin, and differentiation potential.

### Phenotypic characterization of CMSCs and DMSCs

CMSCs and DMSCs at P3-P5 were analyzed by flow cytometry for cell surface markers present on expanded MSCs [2, 9]. More than 90% of CMSCs (Fig 1B and DMSCs (Fig 2B) expressed the MSC markers CD90, CD146, CD166, CD44, CD73 and CD105, while <5% were CD45, CD19 and HLA-DR positive. These findings were consistent with the expression profile stipulated for MSCs. Cells beyond P5 were not analyzed since studies have reported that MSCs undergo cell death or senescence after five passages [4, 21–23].

Given the risk of cross contamination between fetal and maternal cells, firstly we analyzed CMSCs and DMSCs by FISH to verify that pure cell populations had been isolated. CMSCs and DMSCs were isolated from placentae of women delivering a male baby, and approximately 200 cells of each type were analyzed for signals in interphase nuclei using X/Y chromosome probes. CMSCs showed the XY phenotype (Spectrum Green and Orange labelled chromosomes, respectively) and were therefore male (Fig 1Aii). Evaluation of 200 interphase nuclei revealed that CMSCs used in this study had 94% XY (6% XXYY), 100% XY, and 99.5% XY (0.5% XXYY). Two Spectrum Green labelled X chromosomes were visible in DMSCs and therefore DMSCs were female (Fig 2Aii). Evaluation of 200 interphase nuclei showed that DMSCs used in this study had 100% XX, 92.5% XX (7.5% XXXX), and 99% XX (1% XXXX). Cases of tetraploidy were always XXYY from fetal CMSCs and XXXX from maternal DMSCs preparations. Tetraploidy is a common artefact of cell culturing and does not preclude the use of cell preparations for further analysis [24].

Differentiation of expanded CMSCs and DMSCs into osteogenic, adipogenic and chondrogenic lineages was also examined to further verify their *in vitro* MSC properties. Alizarin Red stained calcium deposits indicative of bone formation were visible in CMSCs maintained in osteogenic induction medium (Fig 1Ci). Oil Red O stained lipid droplets were observed around cell nuclei in CMSCs stimulated in adipogenic differentiation medium (Fig 1Cii). CMSCs aggregated into a three-dimensional spherical structure after approximately 24 h stimulation in chondrogenic differentiation medium. Safranin O staining in sections taken from different regions of the cell pellet after 3 weeks stimulation showed the presence of proteoglycans, which are normally secreted into the extracellular matrix by cartilage cells (Fig 1Ciii). Control CMSCs cultures maintained in the appropriate basal medium did not show evidence of differentiation into these lineages (Fig 1Ci–1Ciii insets). DMSCs also differentiated into the osteogenic, adipogenic and chondrogenic mesenchymal lineages (Fig 2Ci, 2Cii and 2Ciii respectively) [13].

### *In vivo* ectopic bone formation assay

**Fig 3:**
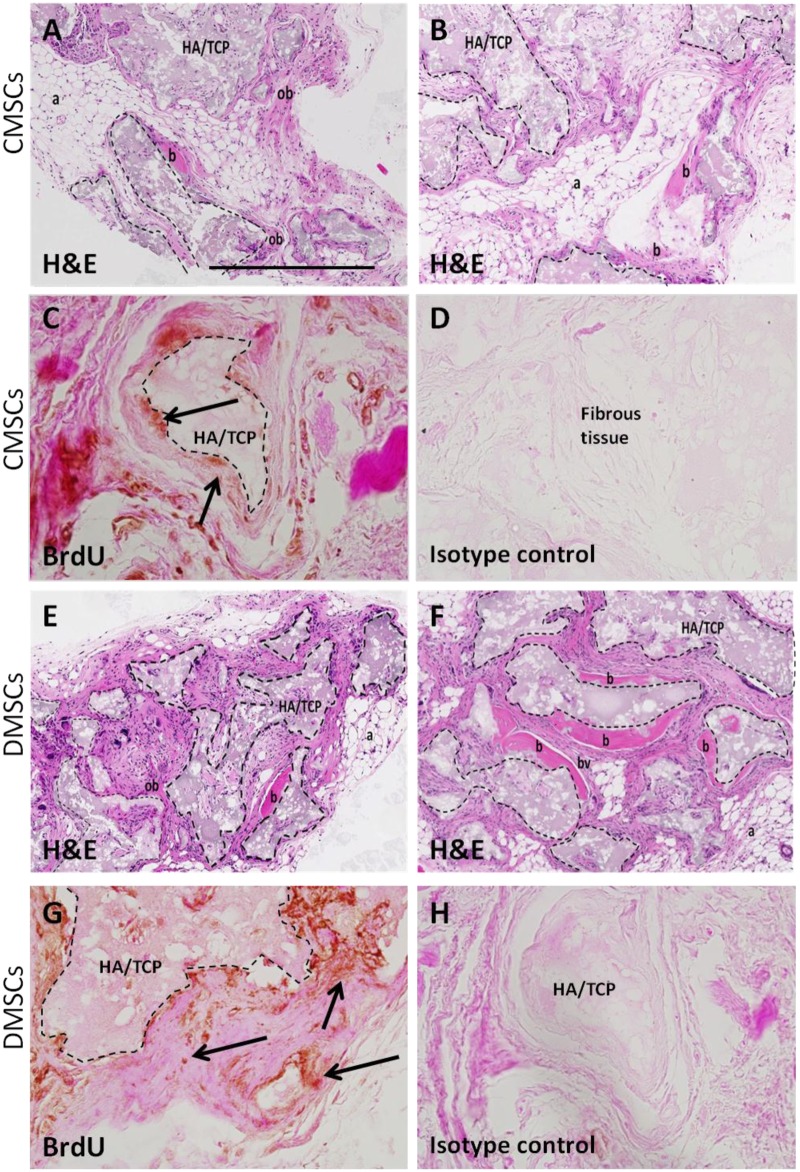
Histology of CMSCs and DMSCs transplants. Cross sections are representative of CMSCs transplants (A-B) and DMSCs transplants (E-F) after 8 weeks stained with Haematoxylin and Eosin (H&E). In the transplant, the HA/TCP carrier surfaces (dashed lines) are lined with new bone formation (b), areas of immature bone (ob) together with the surrounding fibrous and hematopoietic tissue (a) and blood vessel (bv). Representative BrdU staining for localization of implanted CMSCs (C-D) and DMSCs (G-H). BrdU-stained implanted cells were found lining the mineralized matrix (black arrows) and surrounding fibrous tissue. Brown nuclear staining is indicative of DAB reactivity. There was no immunoreactivity present in sections stained with isotype-matched antibodies. HA/TCP: hydroxyapatite/tricalcium phosphate particles. Magnification is 100X and scalebar is 500 μm.

CMSCs and DMSCs (n = 3 per group) were assayed for their capacity to develop bone-like tissue following ectopic transplantation into SCID mice with HA/TCP particles as a vehicle. All implants showed new bone formation throughout the implants. Fig 3A, 3B, 3E and 3F show representative sections of CMSC and DMSC implants, respectively stained with H&E. The H&E staining was performed with bright pink H&E staining is indicative of mineralized tissue [17]. New bone was formed (area of pink staining) in HA/TCP (indicated by dashed lines) and directly interfaces the ceramic surface. The new bone contains osteocytes embedded within the matrix indicating that bone formation was active and progressive. Further histological examination showed that new bone formation was surrounded by the presence of adipocytes (honeycomb-like structures), fibrous tissue, and small blood vessels. Anti-BrdU staining demonstrated the presence of implanted cells associated with areas of mineral formation and areas of dense fibrous tissue formation (Fig 3C, 3D, 3G and 3H). The transplanted CMSCs and DMSCs exhibited the capacity to form mineralized and fibrous tissues *in vivo*.

**Fig 4:**
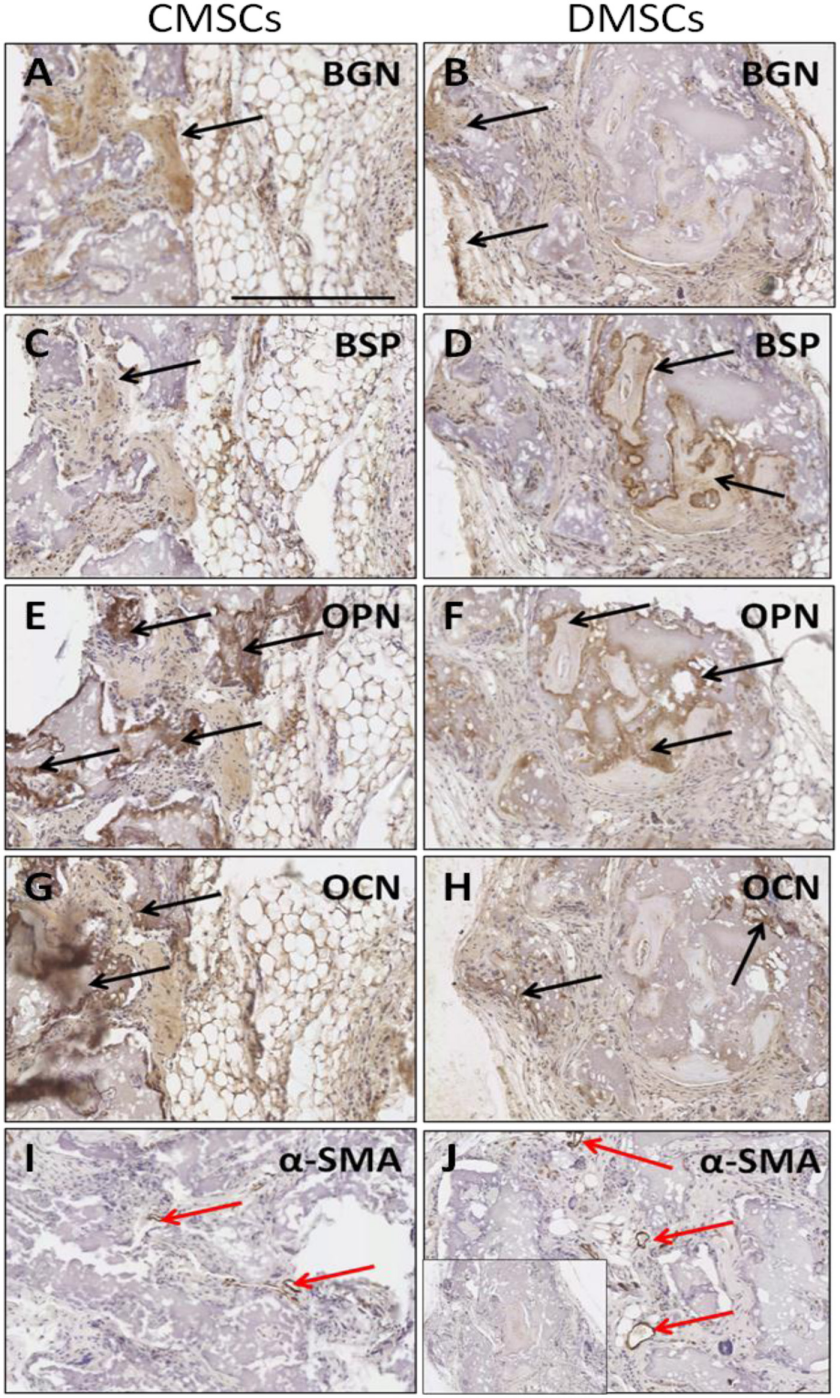
Immunoreactivity of osteogenesis markers after *in vivo* transplantation of primary CMSCs and DMSCs into immunocompromised mice. (A-B) BGN: biglycan expression. (C-D) BSP: bone sialoprotein expression. (E-F) OPN: osteopontin expression. (G-H) OCN: osteocalcin expression. (I-J) α-SMA: alpha-smooth muscle actin as negative control. Inset shows representative control sections stained with isotype-matched antibodies. Colour detection was performed using DAB reaction. Magnification is 200X and scalebar is 300 μm. Black arrow show bone-forming surfaces and red arrows show blood vessels.

Immunohistochemical staining with several osteogenesis markers was performed to confirm that cells with osteocyte properties were formed in the transplants. Implanted CMSCs and DMSCs lining or embedded within the mineralized surfaces expressed the bone-related markers: BSP, OCN, OPN, and BGN (Fig 4A–4H). The reactivity of these markers showed that the implanted CMSCs and DMSCs contributed to the generation of osteogenic cells. In addition, the presence of newly formed vessels in the transplants was also indicated by immunostaining with α-SMA (Fig 4I and 4J). Overall, the data demonstrated the presence of new bone formation with no obvious qualitative differences between CMSCs and DMSCs.

## Discussion

We demonstrated the isolation and expansion of two different MSC populations obtained from human term placenta, fetal-derived CMSCs and maternal-derived DMSCs. Both CMSCs and DMSCs are shown to have typical MSC features: (a) adherence to plastic; (b) ability to differentiate *in vitro* into adipocytes, osteocytes, and chondrocytes; and (c) expression of MSC surface markers (CD90, CD146, CD166, CD44, CD73, and CD105). FISH analysis confirmed that pure populations of fetal CMSCs and maternal DMSCs could be obtained using the isolation methods employed. Thus, the CMSCs and DMSCs adhered to the criteria stipulated for placental MSCs [2, 9].

MSCs from different sources such as adipose tissue, bone marrow, and dental pulp have been subjected to transplantation in the ectopic bone formation assay [14, 15, 25, 26]. This study investigated CMSCs and DMSCs for the first time, and found that HA/TCP implanted with these cells into the subcutaneous space of immunodeficient mice could differentiate into new tissue with characteristics of bone. This indicates that donor MSCs has the capacity for long-term survival and could contribute to the generation of different tissue types *in vivo*. The efficacy of this *in vivo* assay was demonstrated in another study where HA/TCP were implanted with human foreskin fibroblasts and HA/TCP only controls were tested and showed only fibrous tissue growth with no indication of osteogenesis or haematopoiesis [26].

In this study, we set out to confirm that the implanted cells had survived and contributed to tissue formation with BrdU staining. In principle, BrdU stain incorporates into the DNA of dividing cells and diminishes as those cells further divide. Following 8 weeks post-implantation, a proportion of MSCs may have lost their BrdU expression due to their initial proliferation *in vivo*, or due to the physical location within tissue (i.e. osteocytes in lacunae), antigen retrieval and DNA denaturing protocol may not have sufficiently exposed the antigen for its detection by immunohistochemistry. Whilst we acknowledge the limitation of using BrdU to detect implanted cells, this protocol has been widely used and accepted [27–29].

It is also of interest to note that development potential of BMMSCs *in vivo* was similar to CMSCs and DMSCs in which the cells contributed to new bone formation together with surrounding fibrous and adipocytes accumulation [14, 15, 30]. Our findings further support other studies demonstrating placenta-derived MSCs *in vivo* osteogenesis capacity. Intrabone, but not subcutaneous, injection of placenta adherent cells into a mouse model of myeloma-associated bone loss promoted bone formation by stimulating differentiation of the host’s osteoblasts [31]. Furthermore, placenta-derived MSCs grown in a silk fibroin/HA scaffold were transplanted in a rabbit radius defect model and improved bone repair as evidenced by formation of new lamellar bone, trabecular bone and a number of osteoblasts [32]. In addition, there was evidence of angiogenesis with evidence of new blood vessel formation. The presence of newly formed vasculature is in agreement with previous studies that have reported dental pulp stem cells when implanted using the similar *in vivo* mouse model [14, 16]. Another study showed amnion-derived MSCs promoted neovascularisation in an *in vivo* mouse model [33].

Porous HA/TCP showed good tissue tolerance with no immunological or toxic reaction, and that bone tissue could directly deposit upon their surfaces. This property is important for bone graft substitutes because without it, fibrous tissue can intervene at the interface between bone tissue and the graft, and cause loosening of the graft [12, 26]. The choice of osteoinductive biomaterials (HA/TCP) combined with the appropriate choice of animal model are potentially crucial to determine *in vivo* differentiation ability of CMSCs and DMSCs. To conclude, this is the first evidence of *in vivo* differentiation potential of DMSCs and CMSCs following transplantation in the mouse model of ectopic bone formation.

## Conclusions

In this study, we have isolated human CMSCs and DMSCs and both cell types demonstrated the characteristic MSC phenotype. Subcutaneous transplantation of CMSCs and DMSCs embedded in a HA/TCP biomatrix, into a mouse model of ectopic bone formation led to the formation of a bone-like structure. BrdU labelling indicated that transplanted cells were retained in the structure and contributed to tissue formation. Bone-specific markers such as OPN, OCN, BGN, and BSP were present in both transplants without any qualitative difference. These data suggest that human CMSCs and DMSCs have potent *in vivo* bone forming capacity and may be worthwhile candidates for *in vivo* bone tissue repair.
